# Lasting signature of planting year weather on restored grasslands

**DOI:** 10.1038/s41598-020-62123-7

**Published:** 2020-04-06

**Authors:** Anna M. Groves, Jonathan T. Bauer, Lars A. Brudvig

**Affiliations:** 1 Department of Plant Biology, Michigan State University, East Lansing, MI USA; 2 Program in Ecology, Evolutionary Biology, and Behavior, Michigan State University, East Lansing, MI USA; 3 Present Address: Discover Magazine, Kalmbach Media, Waukesha, WI USA; 4 Present Address: Department of Biology, Institute for the Environment and Sustainability, Miami University, Oxford, OH USA

**Keywords:** Community ecology, Restoration ecology

## Abstract

Ecological restoration — the rebuilding of damaged or destroyed ecosystems — is a critical component of conservation efforts, but is hindered by inconsistent, unpredictable outcomes. We investigated a source of this variation that is anecdotally suggested by practitioners, but for which empirical evidence is rare: the weather conditions during the first growing season after planting. The idea of whether natural communities face long-term consequences from conditions even many years in the past, called historical contingency, is a debated idea in ecological research. Using a large dataset (83 sites) across a wide geographic distribution (three states), we find evidence that precipitation and temperatures in the planting year (2–19 years before present) affected the relative dominance of the sown (native target species) and non-sown (mostly non-native) species. We find strong support for lasting planting year weather effects in restored tallgrass prairies, thereby supporting the historically contingent model of community assembly in a real-world setting.

## Introduction

Human impacts on natural ecosystems are a major driver of biodiversity loss worldwide^1,2^, and ecological restoration is an important tool for combating this loss^3–6^. However, outcomes of restoration efforts can vary greatly. Therefore a major challenge for restoration ecology is to determine the causes of this variation and to inform restoration practices that result in more predictable outcomes^7^.

To understand this variation, restoration ecology has drawn on various theories about how ecological communities assemble^7–9^. Deterministic processes may dictate which species are able to establish and persist at a restoration site given the current conditions^10^. For example, edaphic conditions, invasive species pressure, seed mixes sown, landscape context, land-use history, and management decisions can all lead to predictable, repeatable variation in restoration outcomes^11–16^. However, the establishment history of a community can also affect the outcome of assembly^17^. For instance, early arrivers to a community can influence later arrivers through processes such as priority effects^18–20^ and inhibition or facilitation of succession^21,22^, leading to divergent communities even when environmental conditions are otherwise the same. Yet the mechanisms that underlie this model of historically contingent community assembly remain an open question, both in ecology broadly and during restoration specifically^7,17,23,24^.

Restoration outcomes may be historically contingent based on details of the restoration practice used; for example, community composition may reflect identities of species sown to initiate a restoration years later^25,26^. Yet, even under otherwise identical restoration approaches, historically contingent community assembly may result from differences in conditions in the year a restoration project is initiated – in other words, when a community first begins to assemble. In particular, weather conditions after planting may affect restoration outcomes by initiating historically contingent community assembly.

These planting year effects (hereafter, year effects) on restored communities make logical sense, but evidence for year effects in the literature is rare, since most restoration projects and ecological studies are not replicated across planting years^27^. In a recent study, we found that different planting years and experimental precipitation regimes significantly altered the seedling emergence patterns of different species, leading to different plant communities in the first growing season of a restored grassland community^28^. Notably, we found high abundances of non-sown species after high precipitation planting years, which we predicted would result in greater discordance between the composition of the restored plant community and the composition of the seed mix. Important work in Kansas and California grasslands has also shown that planting year^29^ or planting year plus site conditions^30,31^ can affect restoration outcomes. Inter-annual variation in weather conditions has also been shown to affect annual weed communities in agricultural ecosystems^32,33^. This evidence has demonstrated the potential for weather during the planting year to influence community assembly; however, it is unclear whether influences will last for the longer timescales over which restoration success is typically evaluated (e.g., decades). Evidence that demonstrates year effects over longer time scales is even rarer, with one notable exception: in a grassland restoration experiment in the North American Great Plains, herbicide application on an invasive grass followed by sowing of a native grass was highly successful even a decade later when planted in average or above-average precipitation years, but reverted to dominance by the invasive grass when planted in an exceptionally dry year^34,35^.

Although these studies support the conclusion that year effects exist, their relative importance is difficult to study due to the myriad other factors that shape a plant community. For example, the outcomes of grassland restoration are influenced by factors such as soil characteristics, site land-use history, management (e.g. prescribed fire frequency), and the richness and density of the seed mix sown; all of which may have deterministic effects on community assembly^25,36–38^. Additionally, the land-use history and the landscape surrounding a site can introduce additional influence via the quantities and identities of non-sown species present in the local seed bank and dispersing in from surrounding area**s**^39–42^. It therefore remains unknown how important year effects are in context of these other factors known to influence assembly. Moreover, there may be an interaction between weather conditions during planting and these other factors. For instance, the importance of weather during the planting year may decrease over time as sites age; or soil properties, such as water holding capacity, may mediate the effects of variation in planting year precipitation. Restored ecosystems thus present an outstanding opportunity to advance understanding of the relative importance and mechanisms of historical contingency in community assembly, an area of active inquiry^43^.

Finally, it remains unclear exactly what aspects of planting year weather conditions might be most important for subsequent restoration trajectories. Past studies have explored correlations with a limited suite of environmental variables: annual mean temperature, total rainy days^30^; June temperature, June-July precipitation^34^; and deviations from normal growing season (March-October) precipitation and temperature^35^. Stuble *et al*. (2017) and MacDougall *et al*. (2008) notably found no long-term effects of temperature metrics, only precipitation metrics, on plant communities sown in different years^30,35^. However, the experimental work by Groves & Brudvig (2019) suggested that precipitation, though important, is not the only driver of sown community differences after different planting years^28^. Additional factors are known to be important determinants of seeds breaking dormancy, germinating, and establishing. For instance, warmer temperatures, especially in early mornings or springtime, can advance the phenology of some plant species^44^, which can favor certain species over others^45^. Finally, any of these weather conditions may interact with abiotic and biotic conditions at a site to determine restoration outcomes, for instance, if non-native invasive species respond differently to conditions than the target native species^28^. Better understanding of which first year weather conditions influence restoration plantings would explain drivers of variation in restoration outcomes and benefit restoration planning by enabling targeted post-planting restoration actions (i.e., to sites planted in years when such actions would be most beneficial).

In this study, we evaluated the influence and relative importance of year effects resulting from planting year weather conditions for plant communities within restored tallgrass prairies. The tallgrass prairie is nearly extirpated in North America, with only 0.1% of its original extent remaining^46^. After decades of degradation from intensive agriculture and other human activities, prairie species no longer occur in many landscapes, nor do their seeds persist in the seed bank^47^. Therefore, restoration often takes place through seed sowing onto abandoned agricultural lands^48^, which could make the establishment of these restored communities particularly susceptible to abiotic and biotic conditions present during their first growing season.

We surveyed 83 grassland sites, initiated by sowing seeds onto former agricultural lands, to investigate (I) whether planting year conditions related to temperature and precipitation influence the resulting plant community 2–19 years after planting, across a variety of metrics (e.g. species richness and cover of sown and non-sown species), (II) what the relative influence of planting year conditions are compared to other known drivers of variation in restored prairie plant communities (i.e. fire frequency, edaphic conditions, site age, and the richness of the seed mix sown), and (III) whether the influence of planting conditions changes with soil productivity or time since planting. We show that weather conditions have lasting effects on prairie plant communities, particularly through effects on the non-sown species, and that these effects can be of greater magnitude than those of other important drivers of restoration outcomes in this system.

## Results

### Weather conditions in restoration planting years

We looked at a suite of 11 different weather metrics that we hypothesized could impact an assembling plant community, and combined them using a Principal Components Analysis. The first axis, PC1, accounted for 37.6% of the variation in the weather dataset and positively correlated with all metrics that related to higher temperatures. The second axis, PC2 (22.0% of the variation), was positively correlated with all metrics that related to more precipitation. The third axis, PC3, accounted for 14.7% of the variation in the weather dataset and was negatively associated with summer precipitation, spring temperatures, and the maximum number of days without a precipitation event, and positively associated with minimum precipitation in a 30-day period and the mean number of rainy days in a 30-day period. We interpret this axis as associated with mild droughts: low overall precipitation that is consistently distributed throughout the growing season, with the worst dry spell relatively wet and short. The distribution of each variable across sites is provided in Supplemental Fig. S1.

### Effects of planting year on community metrics

**Figure 1:**
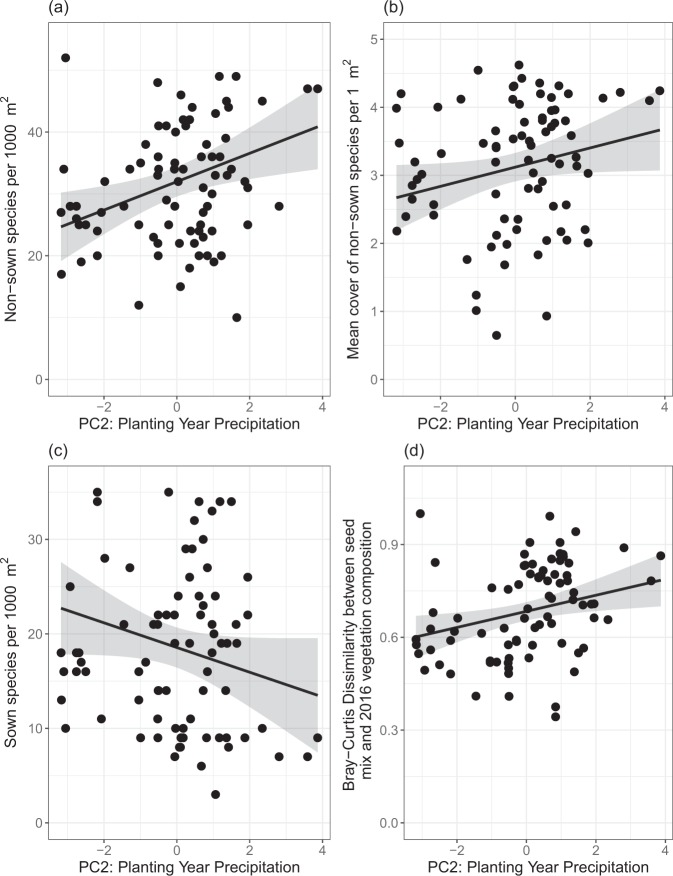
Relationships between planting year weather on restoration outcomes. The effects of planting year weather (PC2: precipitation) are shown on (**a**) the richness of non-sown species (1000 m^2^), (**b**) the cover of non-sown species (mean 1 m^2^ plot), (**c**) the richness of sown species (1000 m^2^), and (**d**) on the Bray-Curtis dissimilarity between the seed mix and the 2016 vegetation composition.

Weather during the planting year affected restored plant communities (Fig. 1). The strongest responses were related to the non-sown species: increased precipitation during the first growing season (PC2) was correlated with increased richness of non-sown species (β = 2.22, F = 7.70, p = 0.0072, Fig. 1a) and marginally correlated with increased cover of non-sown species (β = 0.14, F = 3.77, p = 0.056, Fig. 1b). Increased planting year precipitation (PC2) was also marginally correlated with decreased richness of sown species (β = −1.30, F = 3.40, p = 0.070, Fig. 1c) and was correlated with greater Bray-Curtis dissimilarity between the composition of the seed mix and the 2016 plant community, meaning the communities in prairies planted in wetter years did not resemble their seed mixes as much as prairies planted in drier years (β = 0.026, F = 6.66, p = 0.012, Fig. 1d).

**Figure 2:**
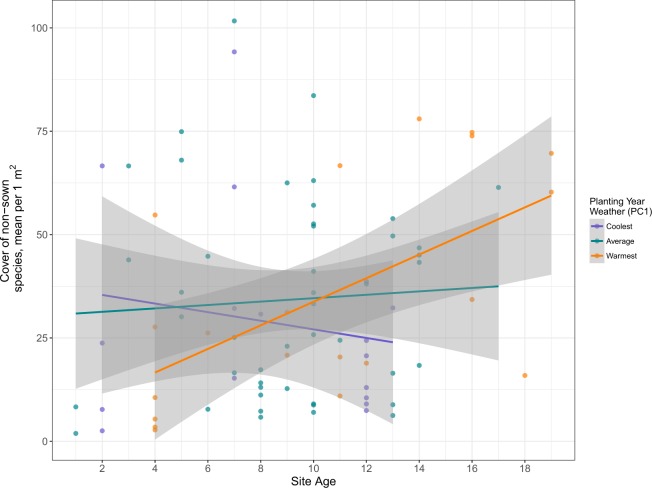
The effect of planting year temperature (weather PC1) on non-sown species cover (mean 1 m^2^ plot) varied depending on site age (p = 0.0027, R^2^ = 0.13). For plotting, sites within 0.75 of one standard deviation from the average PC1 value were assigned the “average” category (22 sites); sites above that threshold were assigned “warmest” (32 sites); sites below the threshold were assigned “coolest” (29 sites).

We also found evidence for a weather by age interaction (Fig. 2), in which cover of non-sown species was positively correlated with age after cooler planting years (PC1), and negatively correlated with age after warmer planting years (PC1) (p = 0.0027, R2 = 0.13).

We found no effects of PC3 (mild droughts in planting year) on any 2016 community metrics, and we found no significant relationships between weather conditions in the first growing season and sown species cover, sown species richness, or total species richness (see Supplementary Table S1 for complete statistical results for all models).

### Relative influence of weather compared to known assembly drivers

**Figure 3:**
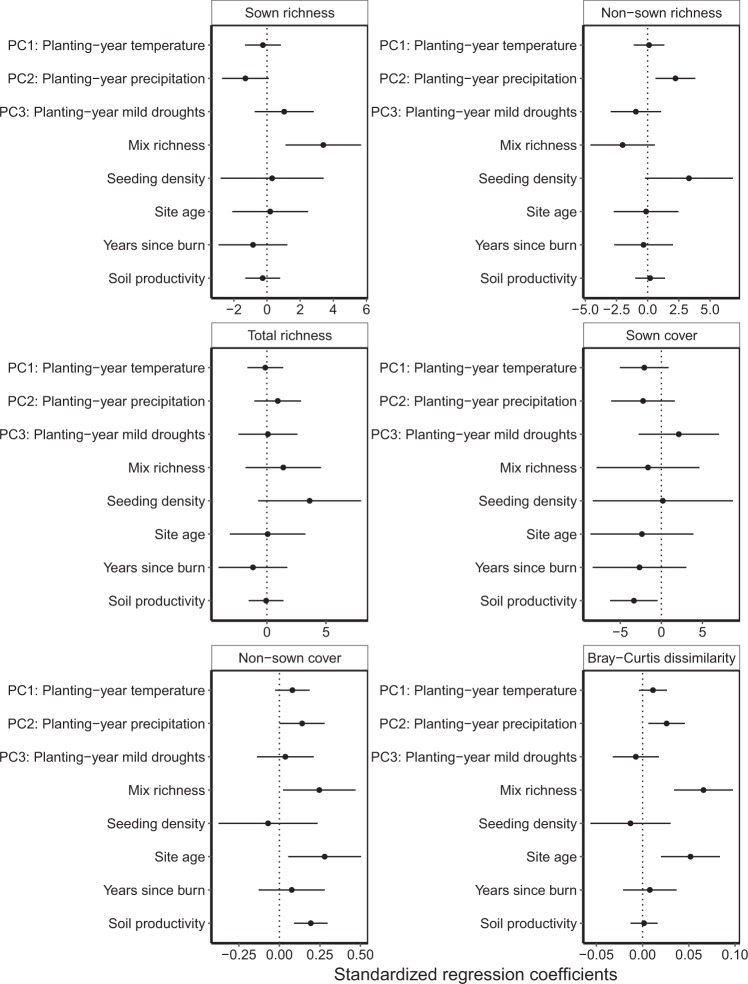
The relative importance of weather conditions and other factors on different measures of restoration outcomes across 83 restored prairies in the Midwestern USA. Each panel shows the standardized regression coefficients with 95% confidence intervals for each continuous predictor. Models included interactions between weather PC1, PC2, PC3 and both site age and soil productivity. Interaction effects and the rest of the full statistical results are reported online in Supplementary Table S1.

The variation in non-sown richness that was explained by planting-year precipitation (PC2, R^2^ = 0.10, Fig. 3) was greater than the variation explained by fire frequency (R^2^ = 0.0011), site age (R^2^ = 0.00016), soil productivity (R^2^ = 0.0016), seed mix richness (R^2^ = 0.036), or seeding density (R^2^ = 0.052).

Non-sown species cover was positively related to, listed in decreasing importance: soil productivity (R^2^ = 0.17, β = 0.19, F = 13.45, p = 0.00049), the interaction between temperature in the first growing season (PC1) and site age (R^2^ = 0.13, β = 0.18, F = 9.73, p = 0.0027), site age (R^2^ = 0.084, β = 0.27, F = 6.02, p = 0.017), seed mix richness (R^2^ = 0.066, β = 0.24, F = 4.64, p = 0.035), and precipitation in the first growing season (PC2, R^2^ = 0.054, β = 0.14, F = 3.77, p = 0.056).

The cover of sown species was not well predicted by our models (total model R^2^ = 0.19), and was best predicted by soil productivity (β = −3.27, F = 5.13, p = 0.027, R^2^ = 0.072). Similarly, sown species richness (total model R^2^ = 0.22) was best predicted by the richness of the seed mix (β = 3.38, F = 8.75, p = 0.0043, R^2^ = 0.12; total model R^2^ = 0.22). We found no significant predictors for overall species richness.

For Bray-Curtis dissimilarity between the seed mix and 2016 community composition, the effect of planting year precipitation (PC2), though significant, was weaker than other predictors. This dissimilarity was better explained by increased seed mix richness (R^2^ = 0.20, β = 0.066, F = 16.78, p = 0.00012) and older site age (R^2^ = 0.13, β = 0.050, F = 10.09, p = 0.0023) than increased planting year precipitation (PC2, R^2^ = 0.092, β = 0.026, F = 6.66, p = 0.012).

## Discussion

Our results show clear evidence for year effects on restored prairie community composition across 83 sites through the effects of planting year weather on the non-sown species. These year effects were predominantly driven by wetter planting years resulting in higher non-sown species richness and marginally higher non-sown cover. Year effects were important across three states, varied soil conditions, and were evident in sites that had been established between two and nineteen years before our study. Additionally, the effect of planting year on non-sown richness was greater than the effects of other known drivers of variation in community assembly in restored prairies (i.e., fire frequency, sown species richness, and soil characteristics). These findings illustrate how specific environmental conditions can lead to historically-contingent community assembly, with persistent and large effects relative to other known assembly drivers.

Sown species richness was marginally decreased following wet planting years (PC2). It is unclear whether this is a result of suppression by the increase in non-sown species that also coincided with wet planting years, or if drier conditions are in fact better for the sown species. We suspect the former, as competition seems a more likely suppressant of sown species establishment than increased water availability. For instance, Groves & Brudvig (2018) found that increased planting-year precipitation increased both sown and non-sown species emergence 3-fold— evidence that sown species do not perform better under drought stress, and that non-sown species increase dramatically under wet conditions^28^.

A mechanism by which early spring precipitation in particular might increase non-sown while decreasing sown richness is that non-sown species in this system are predominantly exotic and have been demonstrated to exhibit advanced phenology compared with the sown native species^49^, and show strong priority effects when competing with native species^50^. Further study and direct experimentation will be needed to conclude whether increased precipitation during a prairie’s first growing season truly increases weed abundance, and whether the resulting competitive pressures stifle development of the sown community for the long term.

We found that the magnitude of the effect of planting year precipitation on non-sown richness was greater than the effects of known major drivers of community assembly in this system: fire frequency, soil productivity, seed mix richness and density, and site age. Though there was some prior evidence for planting year effects, it was unclear whether these would have minor or major influence on restoration outcomes. We show that year effects can be large, but are not consistent across all community metrics (e.g. we found no year effects on total species richness or sown species cover).

We predicted that effects of planting year weather conditions might dampen over time as plantings matured and were subjected to ongoing management and weather conditions (i.e. deterministic assembly dependent on environmental filters^51^); however, we found no negative interactions between weather variables and age since planting, and instead found support for lasting year effects^27^. That said, because weather conditions in the planting year are correlated with site-level climate (see Supplemental Fig. S2 for scatterplots of planting year weather vs. weather in all years since planting), future studies should seek to disentangle this relationship further. Future work should investigate sequences of weather more broadly, including weather leading up to, and after, the planting year. Recent research in restored aridland systems, for example, has shown continued, but decreasing, impacts of warm system precipitation on restoration trajectories after planting and adverse effects of high temperatures years after planting^52^. Additionally, we predicted that effects of planting year conditions, specifically related to precipitation, might be stronger in sites with less productive soils^53^ because of their decreased ability to maintain water balance during times of water stress; however, we found no evidence for an interaction between year effects and soils, which again emphasizes the importance of planting year conditions across a diversity of site conditions^30^.

Because of the increased non-sown and decreased sown richness, wet planting years also correlated with a higher dissimilarity between the seed mix composition and the standing vegetation. These results suggest the need for stronger weed control measures following wet planting years, through more frequent mowing or possibly re-sowing prairie seeds following excessively wet planting years. With short to medium term weather forecasts increasingly available, it may also be possible to prioritize planting during predicted drier years^54,55^ — a suggestion that previously would have seemed counter-intuitive. Our work contrasts work by Bakker *et al*. (2003) and MacDougall *et al*. (2008) which found that a non-sown dominant invader prevailed when restoration occurred in a particularly dry year, which might be a result of climatic differences between our Midwestern sites and the Great Plains, where water may be more limiting, such that drought years more dramatically prevent establishment of sown species^34,35,56–58^.

The impact of planting-year weather patterns on restoration outcomes can also be examined through the lens of future climate projections. In the Midwestern United States, projections are for warmer average temperatures, increased spring precipitation, and decreased summer precipitation^59^. A decrease in summer precipitation may not be as much of a burden to newly planted prairie restorations as an increase in spring precipitation, which in our study was correlated with higher weed pressures that persisted over time, lower richness of sown species, and a vegetation community more dissimilar than the sown seed mix. Our results would therefore suggest an increasing weed burden for prairie restoration practitioners in sites planted under future climate scenarios.

Evidence for lasting planting year effects lends support to historically contingent assembly models^17^, demonstrating that past events can influence the composition of a current community, regardless of deterministic factors like soils or disturbance. Past work has demonstrated the impact of assembly history on experimental communities, including some work in grasslands^20,26,60–63^, and a handful of studies have specifically demonstrated year effects in restored grasslands^28–30,34,35^. Our work builds on this body of research by examining the impact of historical contingency across 83 sites that have been restored across time and space, in context of other factors, and in the pragmatic setting of prairies restored by practitioners.

Our work also contributes important understanding to the question of why restoration and community assembly outcomes vary, showing that both stochastic (weather) and deterministic factors (richness of sown species, soil productivity) factors have roles to play. The marrying of these two principles has been important to community ecology over the past decade^64–67^; we show how they apply to restoration with important implications for interpreting restoration outcomes. In doing so, we illustrate how specific environmental factors can lead to long-lasting historical contingency in how communities assemble.

## Methods

### Study sites

We surveyed 83 restored prairie sites in Illinois, Indiana, and Michigan July-September 2016 that were a maximum of 284 km apart and spanned a 22,800 km^2^ area (see Supplementary Figs. S3 and S4 for photos and a map of study sites). Eighteen of the restored prairie plantings were managed by the McHenry County Conservation District (MCCD) in Illinois. Another 32 sites were managed by The Nature Conservancy’s Kankakee Sands Preserve in Indiana. The final 33 sites were scattered across southwest Michigan, installed by the restoration firm Native Connections (Three Rivers, MI) onto properties under various ownership and management. Prairies ranged in size from 0.2–118 hectares and were established with different seeding techniques (broadcast or drill-seeded), and in different planting seasons (dormant or growing season). Sites also differed in previous land use (soy, corn, winter wheat, hay; some converted directly from production, some left fallow), landscape context (forest, agriculture, or other restored prairie sites), soil characteristics (soil water holding capacity ranged from 0.33–0.80, mean 0.50 percent of total soil weight), and planting year (1998–2015). The majority of sites were managed with prescribed fire, with 75% of sites burned within the past 4 years; 13% of sites had not been burned. Seed mixes sown ranged from a richness of 5 to 221 species (mean 55.9, median 38 species) and a sowing density of 1.6 to 54.1 kg/ha (mean 9.7, median 8.5 kg/ha). Overall, sites varied in site characteristics and management practices both within and across the three states. See Supplementary Fig. S5 for scatter plots of the three PC axis rotations of weather variables against each prairie assembly predictor. Initial statistical models included “state” as a predictor to account for regional differences, but it had no effect on the response variables and was dropped (see “Statistical analyses”).

### Field data collection

We visited the 83 restored prairies between 18 July-16 September 2016. In each site, we established a 50 m transect in a random orientation. For Michigan and Illinois sites, transects were centered on the approximate centroid of the site; for Indiana sites, which were much larger, transects were placed inside the boundary of visible edge effects. At 5- or 10- m intervals (for Michigan and Illinois/Indiana sites, respectively), we placed a 1 m × 1 m quadrat frame on the ground and visually quantified the percent cover of each species present. We surveyed 5 plots per site in Indiana and Illinois, and 10 plots in Michigan (for compatibility with another project outside the scope of this manuscript). In addition, we systematically walked a 20 m x 50 m area centered on the transect and recorded additional species observed. Past studies by our group have demonstrated that this sampling methodology adequately captures variation among sites based on restoration approach (e.g., seed mix richness or composition) and environmental conditions^16,68^. A summary of the species richness and percent cover observed across the sites is available in Supplementary Note.

We collected two soil cores at 5 m intervals along each transect using an Oakfield soil probe (20 total per site, 2 cm × 20 cm soil cores). Soil cores were homogenized at each site. We determined soil water holding capacity by saturating field samples and then drying for 72 hours at 105 °C and calculating the proportional difference in saturated wet weight and oven‐dried weight. Air-dried samples were also analyzed for a variety of soil properties including pH, percent organic matter, percent clay/silt/sand, and soil nutrients (S, P, Ca, Mg, K, Na, B, Fe, Mn, Cu, Zn, and Al) by Brookside Laboratories (New Knoxville, OH, USA). To reduce the potential number of predictor variables, we conducted a principal components analysis on the soils data using prcomp in R with each variable scaled due to large variation the concentrations of soil nutrients. For later analyses, we used the first principal component, which accounted for 40.5% of the variation in the soils data and was positively associated with higher percent organic matter, higher water holding capacity, higher clay content, higher silt content, higher nutrients (except Zn, Fe, and P), and less sand. Hereafter this first PC axis for soils is referred to as “soil productivity.” The full soil variable rotations for PC axes is available in Supplementary Table S2.

### Management histories and historical weather data

We worked with land managers at each site to compile information about the restored prairies, including the date of planting, prescribed fire history, and the relative abundance (by weight) of each species sown in the initial planting. We used 30-arcsec (approximately 800 m) spatially gridded PRISM Climate Data to compile interpolated information on the daily precipitation accumulation and minimum and maximum temperature in the first growing season for each site based on its latitude and longitude^69^. We used these data to calculate cumulative growing degree days (base 10 °C); this temperature-based unit is often used in agronomic systems to predict development of plants and other species^70^. We calculated cumulative precipitation and averages related to temperature and precipitation at various stages throughout the growing season.

### Statistical analysis

To characterize weather conditions at the time of planting we focused on eleven variables that we hypothesized would influence the germination and establishment of plant communities during the first growing season of prairie restoration: spring temperatures (degree day accumulation March 1–June 1), spring precipitation accumulation (March 1–June 1), summer temperatures (degree day accumulation June 1–September 1), summer precipitation accumulation (June 1–September 1), full growing season temperatures (degree day accumulation March 1–September 1), full growing season precipitation accumulation (March 1–September 1), the hottest month (maximum degree days accumulated in a 30-day period), the longest drought (maximum consecutive days without a precipitation event), the driest month (minimum precipitation in a 30-day period, in mm), average low temperature (March 1–September 1, °C), and the average monthly precipitation (March 1–September 1, mm). We focused on this set of variables owing to the reported roles of temperature and precipitation for plant establishment dynamics^28,71^ and for year effects broadly^30,34,35^. Because many of these variables were correlated (see Supplementary Fig. S6 for scatter plots and pairwise correlations between weather variables), we conducted a Principal Components Analysis to determine composite metrics with which to test our hypotheses (see Supplementary Table S3 for weather variable rotations for PC axes 1, 2, and 3). We focused on the first three axes (out of eleven total) that together accounted for 74.2% of the variation in weather data.

We used a series of general linear models to test for an effect of planting year weather conditions (PC1, PC2, and PC3) on the present-day community. In addition to the three weather PCs, models included the richness of the seed mix, the seeding density (kg/ha), the age of the site in 2016, the frequency of fire (characterized as the years since the last prescribed burn), and soil productivity. None of these variables were colinear, with each correlation coefficient (r) < 0.4 (44). See Supplementary Fig. S7 for scatter plots and pairwise correlations between prairie site variables. Initial models included state (Michigan, Illinois, Indiana) as a factor to account for any regional differences in general climate or other factors not otherwise represented by other model variables; however, there was no effect of state on any response variables and, since this was not a hypothesis we were testing, we did not include this in our final models. Each model also included interactions between PC1, PC2, and PC3 and both site age and soil productivity to test if the effects of planting year weather diminish over time or if these effects are mediated by soil properties.

Finally, we considered response variables representing typical prairie restoration goals: namely, improving sown species richness and cover and minimizing non-sown species richness and cover. We therefore ran six models, predicting the richness of sown, non-sown, and total species at the site level (20 m × 50 m survey area); the cover of sown and non-sown species at the mean 1 m^2^ plot level; and the Bray-Curtis dissimilarity between the composition of the seed mix sown and the vegetation at the site in 2016. We compared R^2^ values of predictors in the models to compare their relative influence on planting outcomes.

## Supplementary information

Supplementary Materials.

## Data Availability

The datasets generated and analyzed, and the R code used in this study are available in the Dryad repository, 10.5061/dryad.6q573n5vt.
