# Supplementary material for: Lasting signature of planting year weather on restored grasslands
Source: Sci Rep. 2020 Apr 6;10:5953. doi: 10.1038/s41598-020-62123-7 (PMC7136215; doi:10.1038/s41598-020-62123-7)
Supplement: Supplementary file 1 — Supplementary Materials. [file 41598_2020_62123_MOESM1_ESM.pdf]

SUPPLEMENTARY MATERIALS

MS: SREP-19-28398A
Title: Lasting signature of planting year weather on restored grasslands
Authors: Anna M. Groves*, Jonathan T. Bauer and Lars A. Brudvig
*Corresponding author, groves.anna@gmail.com

Contents:

Supplementary Figure S1. Distributions of weather and site condition variables across sites.
Supplementary Figure S2. Correlations between weather in the planting year (January 1 to December 31 for the year of the site's first growing season) and the weather across all years (January 1 of planting year to December 31, 2015) for each prairie site.
Supplementary Figure S3. Photos of restored prairies.
Supplementary Figure S4. Map of study sites.
Supplementary Figure S5. Scatter plots of the three PC axis rotations of weather variables against each prairie assembly predictor
Supplementary Figure S6. Scatter plots and pairwise correlations (r) between weather variables.
Supplementary Figure S7. Scatter plots and pairwise correlations (r) between prairie site variables.

Supplementary Note.

Supplementary Table S1. Complete statistical results for models analyzing the relative importance of weather conditions and other factors on prairie restoration outcomes.
Supplementary Table S2. Soil variable rotations for PC axes.
Supplementary Table S3. Weather variable rotations for PC axes 1, 2, and 3

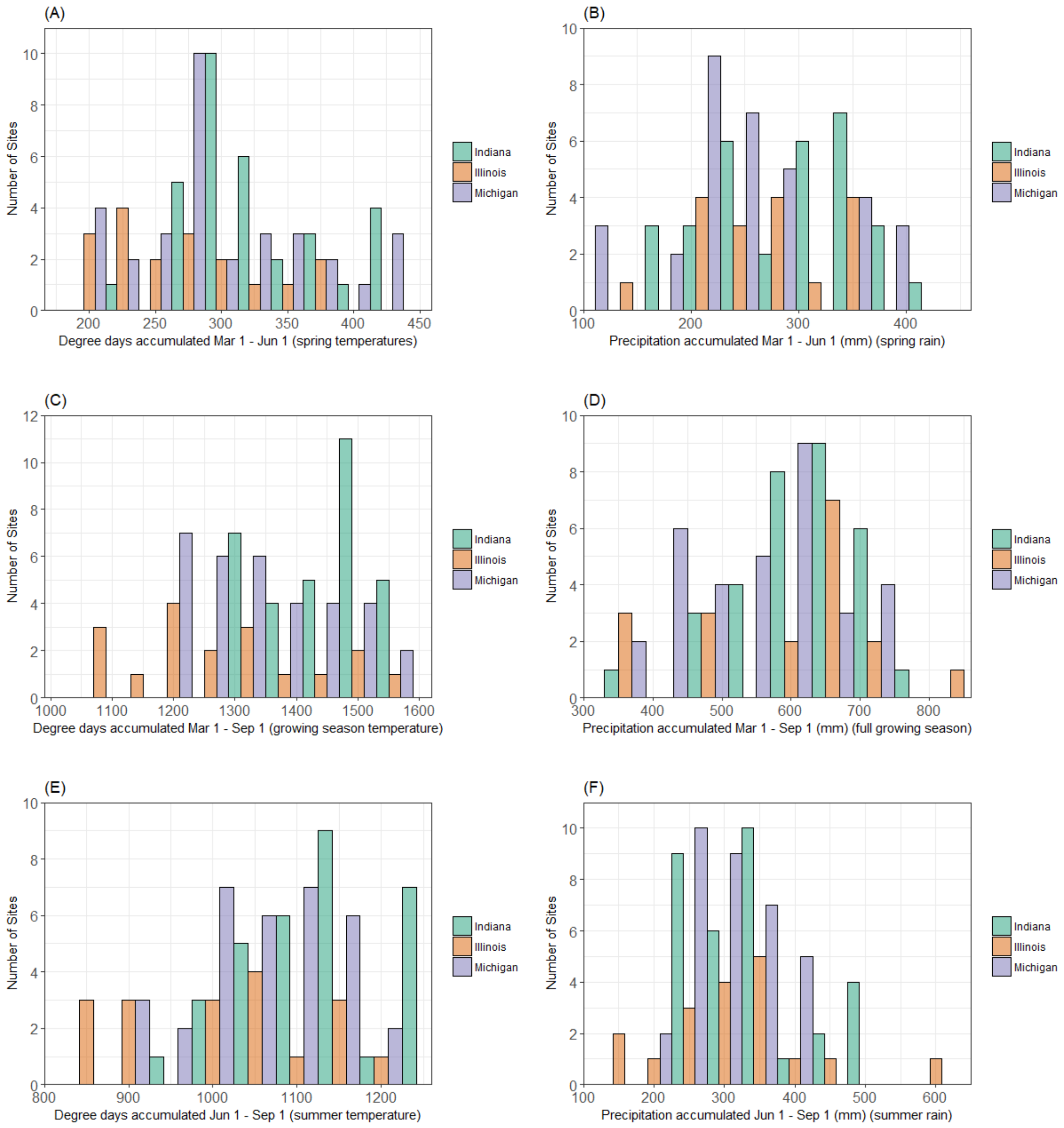

Supplementary Figure S1. Distributions of weather and site condition variables across sites.

Supplementary Figure S1 (cont'd)

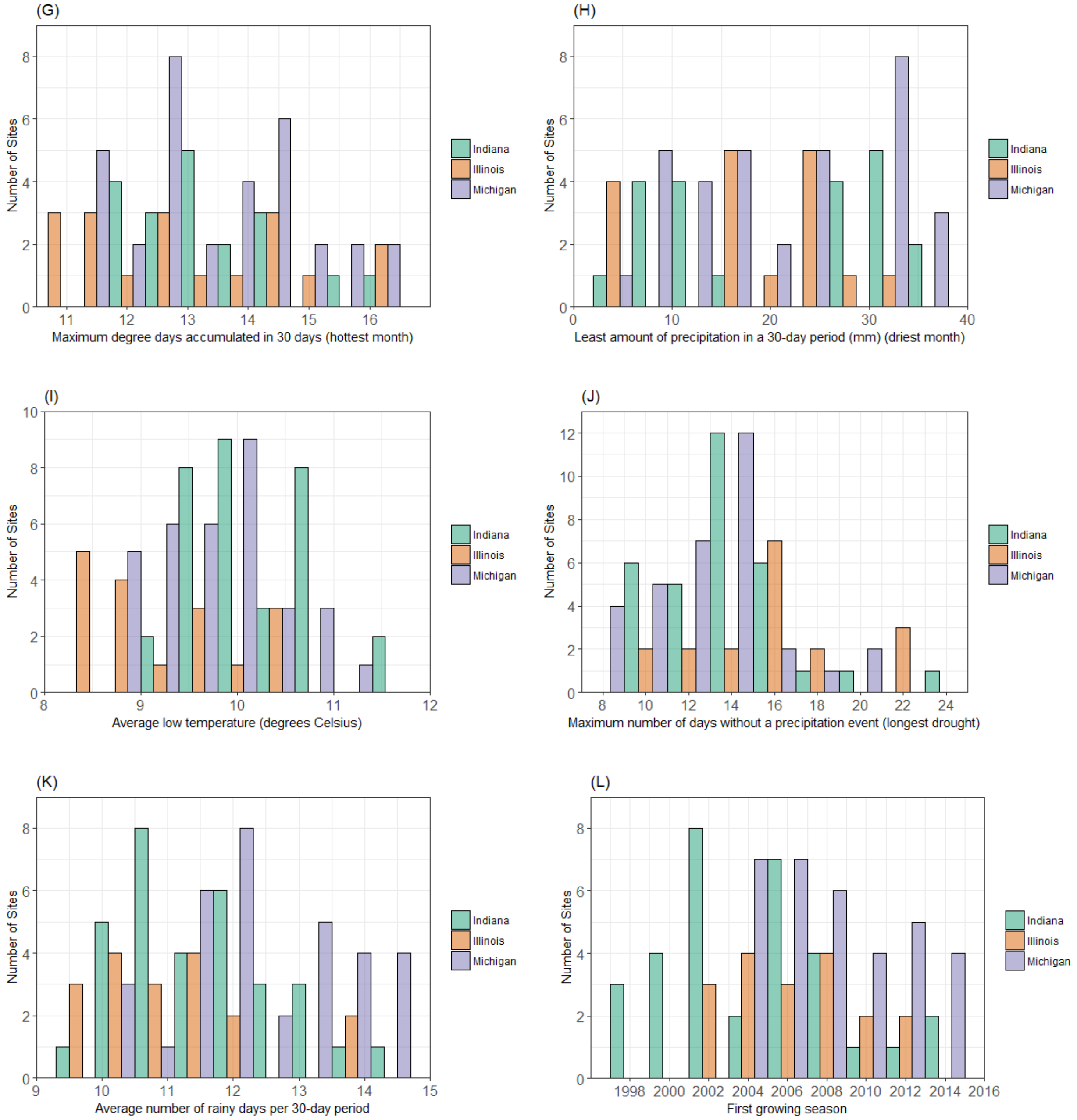

Supplementary Figure S1 (cont'd)

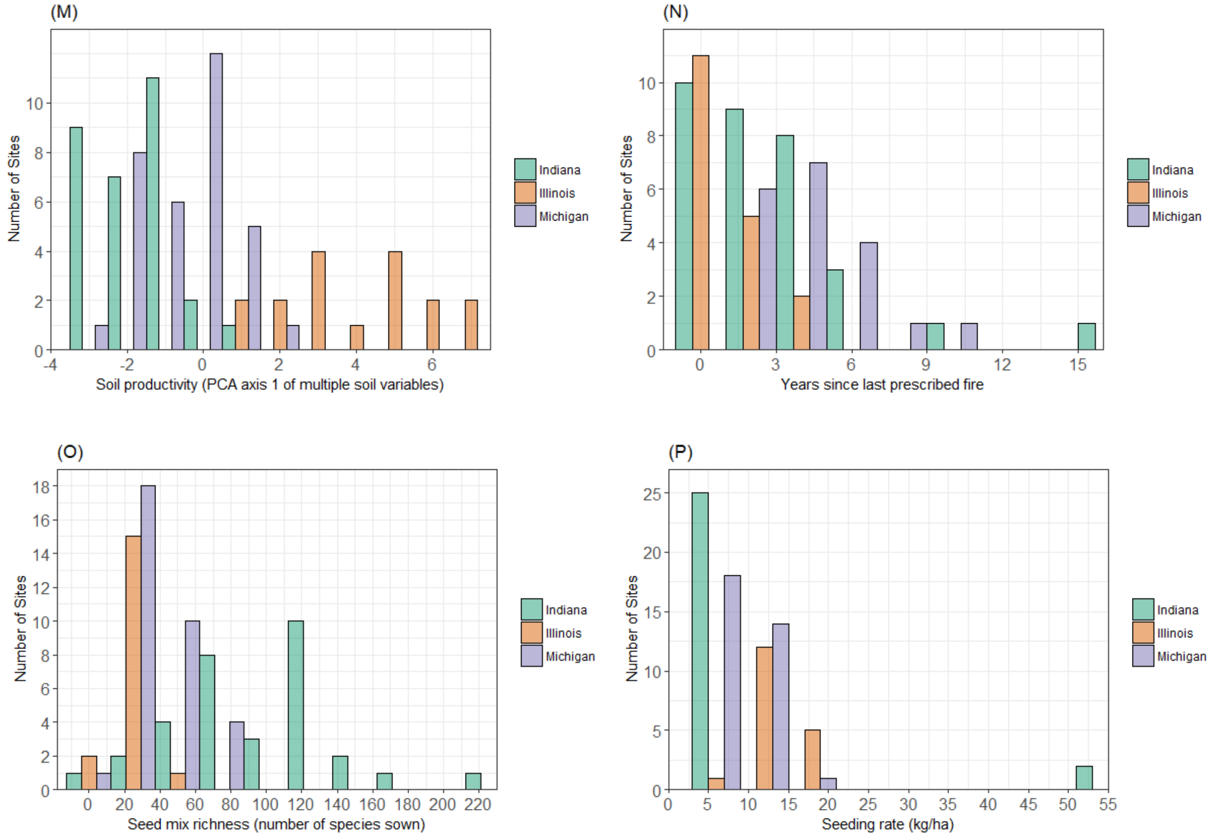

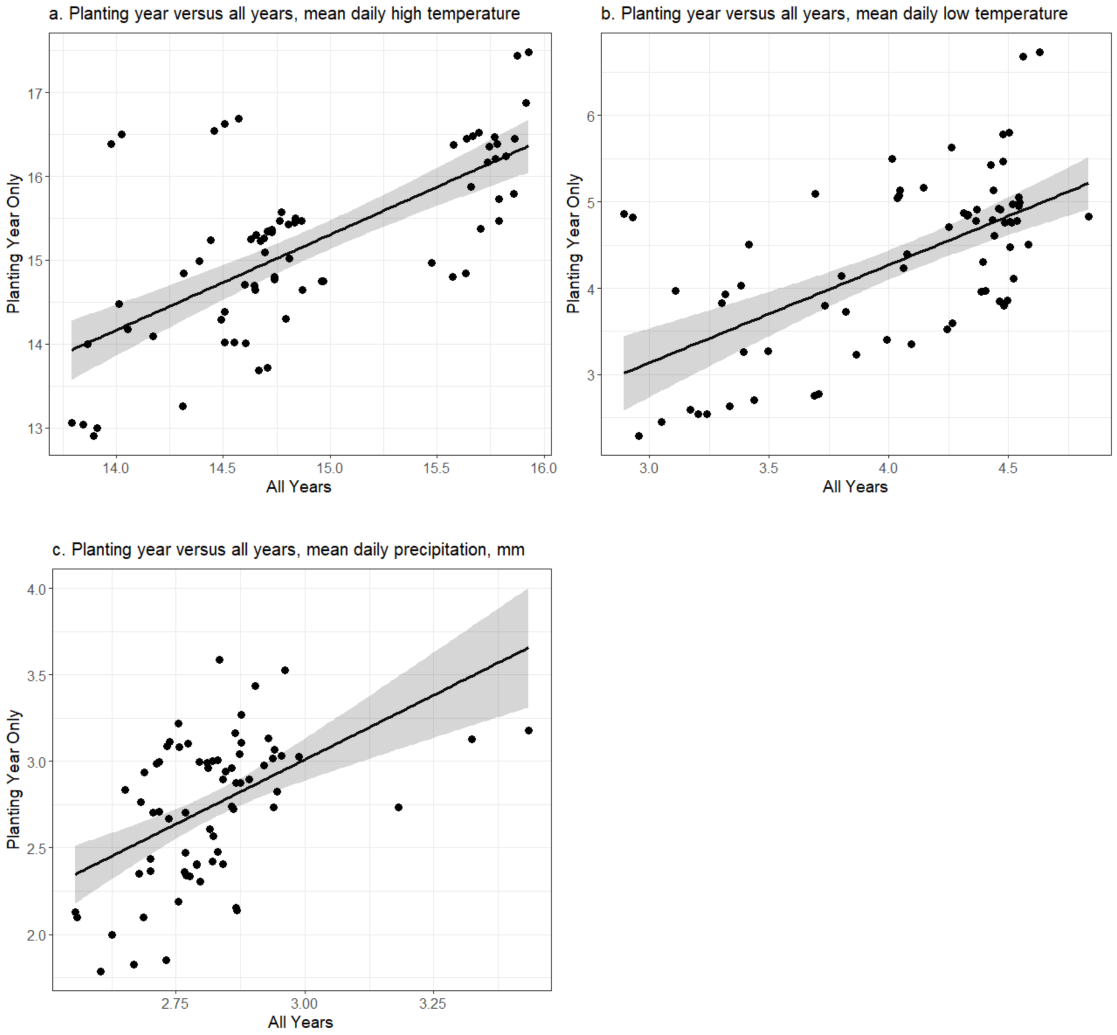

Supplementary Figure S2. Correlations between weather in the planting year (January 1 to December 31 for the year of the site's first growing season) and the weather across all years (January 1 of planting year to December 31, 2015) for each prairie site. All three metrics examined were correlated, but weather across all years accounted for less than half the variation in planting year conditions for (a) mean daily high temperature (adjusted $R^2 = 0.45$), (b) mean daily low temperature ($R^2 = 0.34$), and (c) mean daily precipitation ($R^2 = 0.24$). The shaded area is the 95 percent confidence interval on the general linear model of climate on planting year weather.

(a)

(b)

(c)

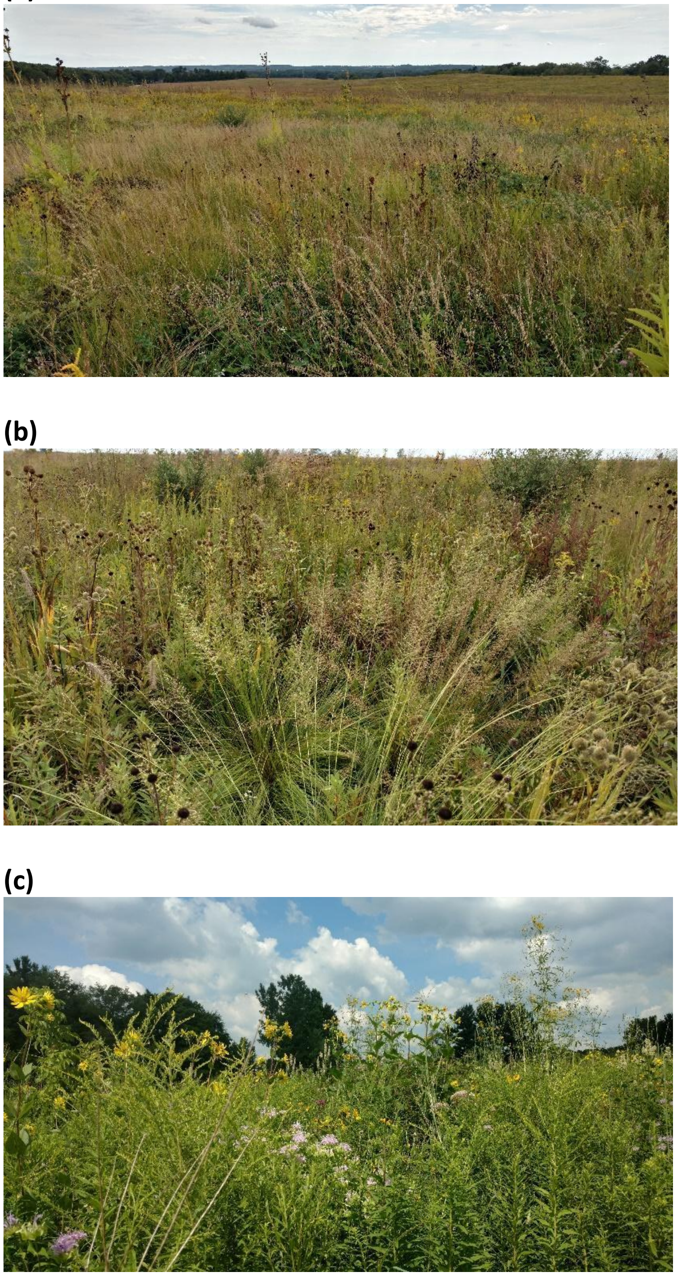

Supplementary Figure S3. Photos of restored prairies. (a) Marengo Ridge (planted 2009), McHenry County, Illinois on September 13, 2016. (b) Kankakee Sands Preserve unit J (planted 2009), Newton County, Indiana on September 8, 2016, and (c) Ed Lowe Foundation (planted 2011), Cass County, Michigan on July 27, 2016. All photos taken by Anna Groves.

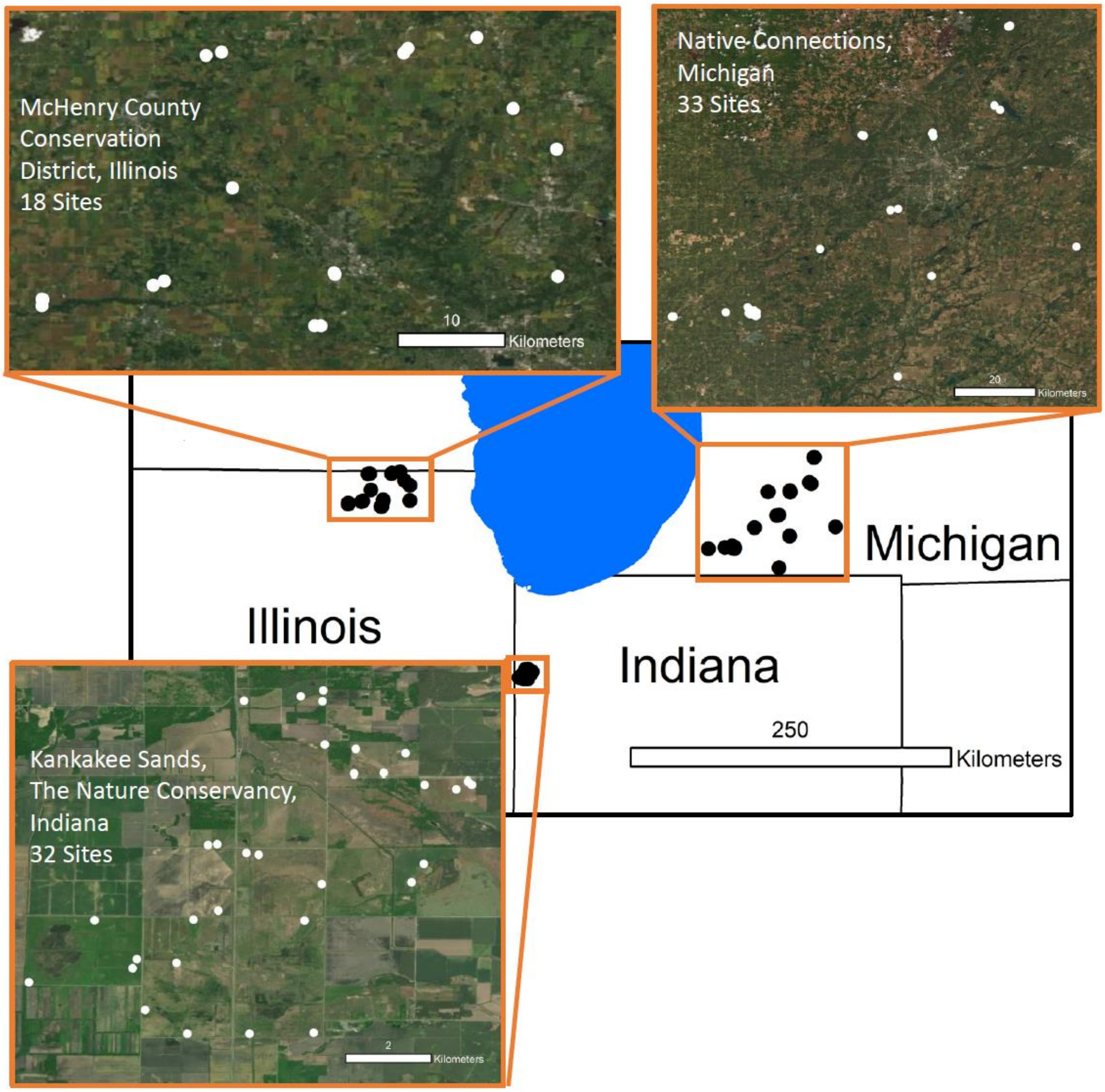

Supplementary Figure S4. Map of study sites. Map images created using ArcGIS® software by Esri. ArcGIS® and ArcMap™ are the intellectual property of Esri and are used herein under license. Copyright © Esri. All rights reserved. Basemap satellite images by Esri, DigitalGlobe, GeoEye, Earthstar Geographics, CNES/Airbus DS, USDA, USGS, AeroGRID, IGN, and the GIS User Community.

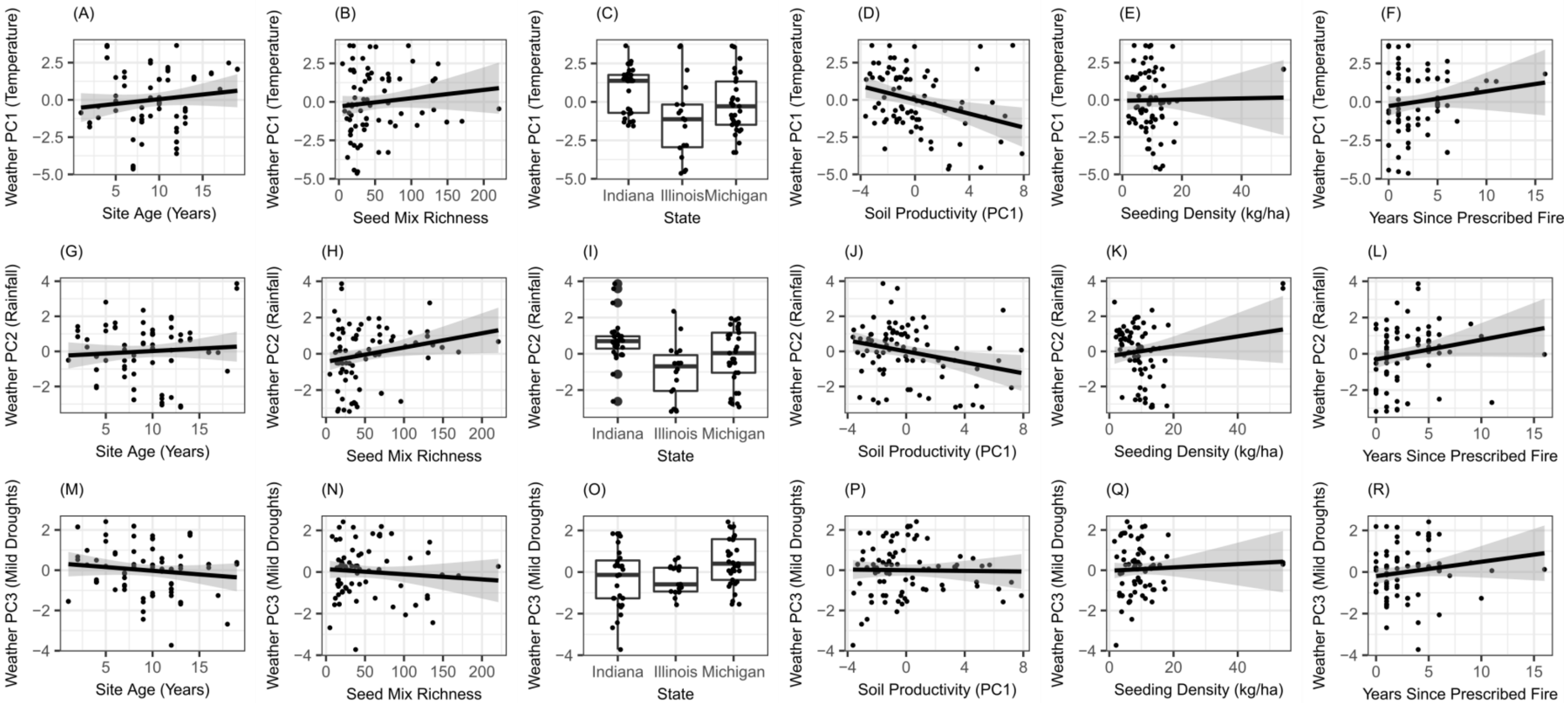

Supplementary Figure S5. Scatter plots of the three PC axis rotations of weather variables against each prairie assembly predictor. PC axis 1 (temperature) is plotted against (A) site age, (B) seed mix richness, (C) state, (D), soil productivity, (E) seeding rate, and (F) years since last prescribed fire; PC axis 2 (rainfall) is likewise plotted against (G) site age, (H) seed mix richness, (I) state, (J), soil productivity, (K) seeding rate, and (L) years since last prescribed fire; and PC axis 3 (mild droughts) is plotted against (M) site age, (N) seed mix richness, (O) state, (P), soil productivity, (Q) seeding rate, and (R) years since last prescribed fire.

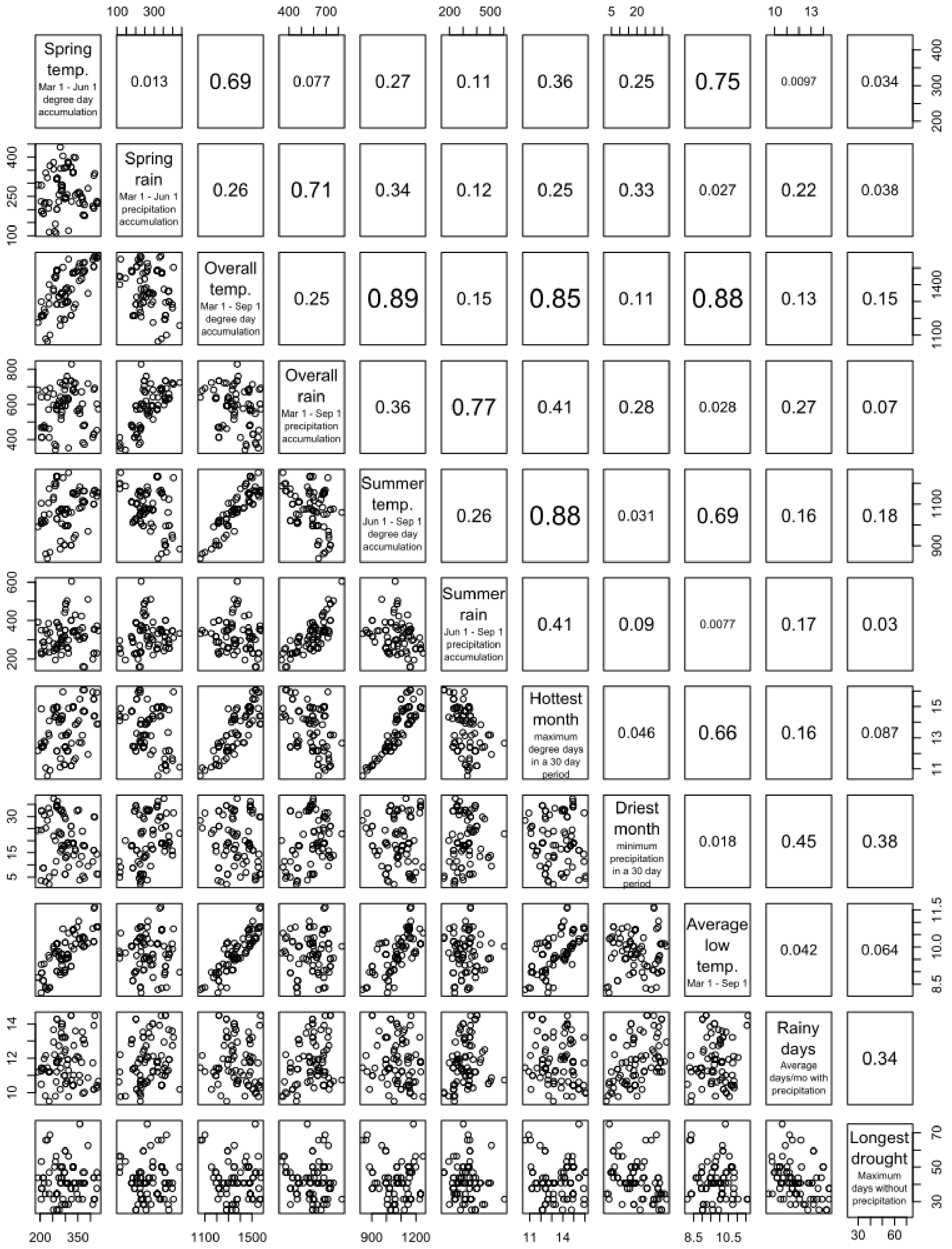

Supplementary Figure S6. Scatter plots and pairwise correlations (r) between weather variables.

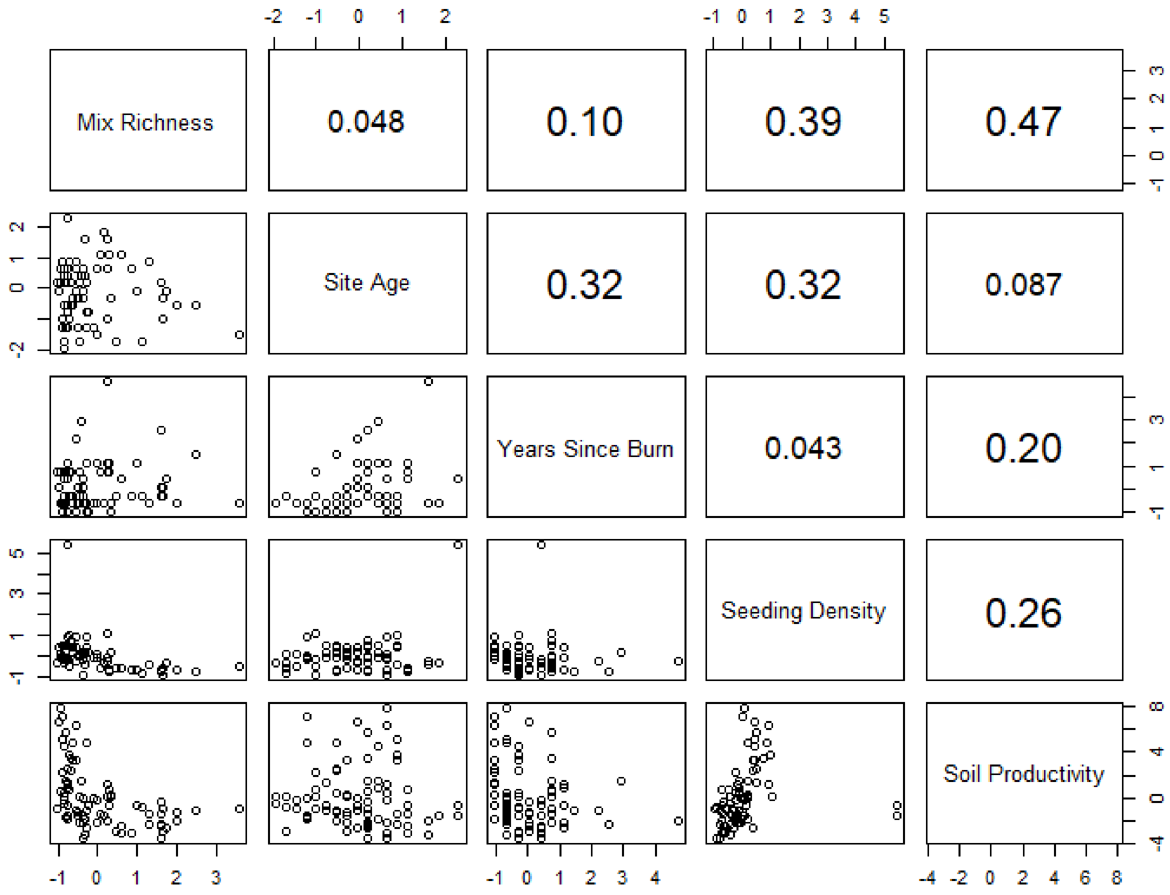

Supplementary Figure S7. Scatter plots and pairwise correlations (r) between prairie site variables.

Supplementary Note.

Total observed species richness in the 83 prairie sites ranged from 23-73 species per site (mean 50), and at the plot (1 m^2) scale, species richness ranged from 4-18 (mean 11). Sown species richness ranged from 3-35 (mean 18) species at the field scale and from 0-28 (mean 11) at the 1 m^2 scale. The average percent cover of sown species per plot ranged from 0-106% (mean 51%), while mean non-sown species cover ranged from 2-102% (mean 33%); overlapping individuals allow for sums greater than 100% per plot. The average dissimilarity between the composition of the seed mix and the standing community was 0.69 (range 0.34-1.00); between 0 and 88% of species sown were present at each site.

Supplementary Table S1. Complete statistical results for models analyzing the relative importance of weather conditions and other factors on prairie restoration outcomes. Asterisks denote statistical significance at p<0.05.

A. Species richness (1000 m²)				*Model R^2=0.24*
Predictor	*β*	*F*	*p-value*	*R^2*
Weather PC1 (temperature)	-0.11	0.02	0.89	<0.01
Weather PC2 (precipitation)	0.92	0.86	0.36	<0.01
Weather PC3	0.06	<0.01	0.96	0.05
Seed mix richness	1.36	0.72	0.40	<0.01
Seeding density	3.40	2.65	0.11	0.03
Years since last fire	-1.23	0.71	0.40	0.02
Soil productivity	-0.04	<0.01	0.96	<0.01
Site age	0.16	0.01	0.92	<0.01
Weather PC1 (temperature) X Site Age	0.42	0.27	0.61	<0.01
Weather PC2 (precipitation) X Site Age	-1.36	1.18	0.28	0.03
Weather PC3 X Site Age	0.02	<0.01	0.99	0.03
Weather PC1 (temperature) X Soils	0.39	2.71	0.10	0.10
Weather PC2 (precipitation) X Soils	0.33	0.82	0.37	0.01
Weather PC3 X Soils	<0.01	<0.01	0.99	0.02
B. Sown species richness (1000 m²)				*Model R^2=0.22*
Predictor	*β*	*F*	*p-value*	*R^2*
Weather PC1 (temperature)	-0.23	0.17	0.68	<0.01
Weather PC2 (precipitation)	-1.30	3.40	0.07	0.05
Weather PC3	1.04	1.35	0.25	0.02
Seed mix richness	3.38	8.75	<0.01**	0.12
Seeding density	0.20	0.02	0.89	<0.01
Years since last fire	-0.91	0.76	0.39	0.01
Soil productivity	-0.23	0.19	0.66	<0.01
Site age	0.29	0.07	0.80	<0.01
Weather PC1 (temperature) X Site Age	-0.28	0.22	0.64	<0.01
Weather PC2 (precipitation) X Site Age	0.38	0.18	0.67	<0.01
Weather PC3 X Site Age	-0.53	0.30	0.59	<0.01
Weather PC1 (temperature) X Soils	0.09	0.28	0.60	<0.01
Weather PC2 (precipitation) X Soils	0.24	0.88	0.35	0.01
Weather PC3 X Soils	0.06	0.02	0.89	<0.01
C. Non-sown species richness (1000 m²)				*Model R^2=0.24*
Predictor	*β*	*F*	*p-value*	*R^2*
Weather PC1 (temperature)	0.12	0.04	0.85	<0.01
Weather PC2 (precipitation)	2.22	7.70	<0.01**	0.10
Weather PC3	-0.98	0.94	0.34	0.01
Seed mix richness	-2.02	2.44	0.12	0.04
Seeding density	3.20	3.59	0.06	0.05
Years since last fire	-0.32	0.07	0.79	<0.01
Soil productivity	0.20	0.11	0.74	<0.01

Supplementary Table S1 (cont'd)

Site age	-0.13	0.01	0.92	<0.01
Weather PC1 (temperature) X Site Age	0.70	1.12	0.29	0.02
Weather PC2 (precipitation) X Site Age	-1.74	2.98	0.09	0.04
Weather PC3 X Site Age	0.54	0.25	0.62	<0.01
Weather PC1 (temperature) X Soils	0.30	2.48	0.12	0.04
Weather PC2 (precipitation) X Soils	0.09	0.08	0.77	<0.01
Weather PC3 X Soils	-0.05	0.01	0.92	<0.01

D. Cover of sown species (1 m^2 plots)				*Model R^2=0.19*
Predictor	*β*	*F*	*p-value*	*R^2*
Weather PC1 (temperature)	-2.11	1.99	0.16	0.03
Weather PC2 (precipitation)	-2.16	1.23	0.27	0.02
Weather PC3	2.13	0.75	0.39	0.01
Seed mix richness	-1.62	0.27	0.61	<0.01
Seeding density	0.07	<0.01	0.99	<0.01
Years since last fire	-2.77	0.94	0.34	0.01
Soil productivity	-3.27	5.13	0.03*	0.07
Site age	-2.10	0.46	0.50	<0.01
Weather PC1 (temperature) X Site Age	-2.56	2.57	0.11	0.04
Weather PC2 (precipitation) X Site Age	1.54	0.40	0.53	<0.01
Weather PC3 X Site Age	1.29	0.23	0.63	<0.01
Weather PC1 (temperature) X Soils	-0.15	0.10	0.76	<0.01
Weather PC2 (precipitation) X Soils	0.55	0.60	0.44	0.01
Weather PC3 X Soils	-0.28	0.06	0.81	<0.01

E. Cover of non-sown species (1 m^2 plots) (log transformed)				*Model R^2=0.40*
Predictor	*β*	*F*	*p-value*	*R^2*
Weather PC1 (temperature)	0.09	2.50	0.12	0.04
Weather PC2 (precipitation)	0.14	3.77	0.06	0.05
Weather PC3	0.03	0.13	0.72	<0.01
Seed mix richness	0.24	4.64	0.04*	0.07
Seeding density	-0.06	0.18	0.67	<0.01
Years since last fire	0.08	0.56	0.46	<0.01
Soil productivity	0.19	13.45	<0.01***	0.17
Site age	0.27	6.02	0.02*	0.08
Weather PC1 (temperature) X Site Age	0.18	9.73	<0.01**	0.13
Weather PC2 (precipitation) X Site Age	-0.04	0.24	0.62	<0.01
Weather PC3 X Site Age	-0.03	0.07	0.79	<0.01
Weather PC1 (temperature) X Soils	0.01	0.53	0.47	<0.01
Weather PC2 (precipitation) X Soils	-0.02	0.60	0.44	<0.01
Weather PC3 X Soils	<0.01	<0.01	0.98	<0.01

F. Bray-Curtis dissimilarity between seed mix and 2016 vegetation				*Model R^2=0.54*
Predictor	*β*	*F*	*p-value*	*R^2*
Weather PC1 (temperature)	0.01	2.40	0.13	0.04
Weather PC2 (precipitation)	0.03	6.66	0.01*	0.09
Weather PC3	-<0.01	0.36	0.55	<0.01

Seed mix richness	0.07	16.78	<0.01***	0.20

Supplementary Table S1 (cont'd)

Seeding density	-0.01	0.35	0.55	<0.01
Years since last fire	<0.01	0.31	0.58	<0.01
Soil productivity	0.01	0.02	0.88	<0.01
Site age	0.05	10.09	<0.01**	0.13
Weather PC1 (temperature) X Site Age	<0.01	0.70	0.41	0.02
Weather PC2 (precipitation) X Site Age	-<0.01	0.19	0.67	0.03
Weather PC3 X Site Age	<0.01	<0.01	0.98	<0.01
Weather PC1 (temperature) X Soils	<0.01	1.47	0.23	0.02
Weather PC2 (precipitation) X Soils	-<0.01	1.69	0.20	0.03
Weather PC3 X Soils	-<0.01	0.06	0.81	<0.01

Supplementary Table S2. Soil variable rotations for PC axes. PC axis 1 accounted for 40.5% of the variation in the soils data. PC axis 2 accounted for 15.4%, PC axis 3 accounted for 12.0%.

	PC1	PC2	PC3
pH	0.15	-0.34	-0.24
Soil organic matter	0.28	-0.10	0.35
S (ppm)	0.08	0.23	0.46
P (mg/kg)	-0.14	0.18	0.37
Ca (mg/kg)	0.31	-0.19	0.11
Mg (mg/kg)	0.33	-0.10	0.01
K (mg/kg)	0.23	0.15	0.11
Na (mg/kg)	0.17	-0.19	0.10
B (mg/kg half detection)	0.28	-0.22	0.07
Fe (mg/kg)	-0.13	-0.28	0.24
Mn (mg/kg)	0.22	0.14	-0.30
Cu (mg/kg half detection)	0.29	-0.03	-0.02
Zn (mg/kg)	-0.03	-0.34	0.30
Al (mg/kg)	0.05	0.52	0.24
Water holding capacity	0.29	-0.09	0.27
% Clay	0.31	0.07	-0.16
% Silt	0.28	0.29	-0.11
% Sand	-0.31	-0.24	0.14

Supplementary Table S3. Weather variable rotations for PC axes 1, 2, and 3. Values >|0.3| are denoted in bold for emphasis. PC axis 1 accounted for 37.5% of the variation in the weather data, PC axis 2 accounted for 22.0%, and PC axis 3 accounted for 14.7%.

	PC1 (temperature)	PC2 (precip-itation)	PC3 (mild droughts)
Spring weather (March 1 – June 1)			
Degree day accumulation	0.28	0.28	**-0.37**
Precipitation accumulation	-0.20	**0.37**	-0.06
Summer weather (June 1 - September 1)			
Degree day accumulation	**0.44**	0.06	0.17
Precipitation accumulation	-0.19	**0.35**	**-0.33**
Total growing season weather (March 1 – September 1)			
Degree day accumulation	**0.47**	0.17	-0.05
Precipitation accumulation	-0.25	**0.49**	-0.24
Mean low temp	**0.39**	0.33	-0.15
Growing season extremes			
Maximum degree days in a 30-day period	**0.45**	0.02	0.13
Minimum precipitation in a 30-day period	-0.10	**0.32**	**0.52**
Maximum number of days without a precipitation event	-0.04	-0.26	**-0.48**
Mean number of rainy days per 30-day period	-0.12	**0.34**	**0.35**
